# Smart mating: the cognitive ability of females influences their preference for male cognitive ability

**DOI:** 10.1093/beheco/arab052

**Published:** 2021-06-22

**Authors:** Náyade Álvarez-Quintero, Alberto Velando, Sin-Yeon Kim

**Affiliations:** Centro de Investigación Mariña, Universidade de Vigo, Grupo Ecoloxía Animal, Torre CACTI, Campus de Vigo, 36310 Vigo, Spain

**Keywords:** cognition, *Gasterosteus aculeatus*, mate-choice, secondary sexual traits, sexual selection

## Abstract

Cognitive abilities may be crucial for individuals to respond appropriately to their social and natural environment, thereby increasing fitness. However, the role of cognitive traits in sexual selection has received relatively little attention. Here, we studied 1) whether male secondary sexual traits (colour, courtship, and nest) reflect their cognitive ability, 2) whether females choose mates based on males' and their own cognitive abilities, and 3) how the interplay between secondary sexual traits and cognitive ability determines male attractiveness in the three-spined stickleback (*Gasterosteus aculetaus*). For this, we first evaluated the cognitive ability of sexually mature males and females in a detour-reaching task. Then, female preference was repeatedly assessed in a dichotomous-choice test, where the female was exposed to two males with contrasting performances (relatively good and bad) in the detour-reaching task. Female preference for better performing males was affected by the female's own cognitive ability. Females with relatively medium-low cognitive ability preferred males with high ability, whereas females with high ability showed no preference. We also found that males with higher cognitive abilities built more elaborated nests, but showed weaker red nuptial colouration. To our knowledge, this is among the first results that illustrate how cognitive traits of both sexes influence female mate preference, which has implications for the strength and direction of sexual selection.

## Teaser

Cognition may aid in the development of behavioral sexual displays, thereby contributing to mating success. In this study, female sticklebacks with relatively low cognitive ability preferred mating with males with better cognitive ability, while those with high ability did not have preference for smart males. Male’s cognitive ability was positively related to nest structure but negatively related to nuptial color. Our results show the complex interplay between secondary sexual traits and cognitive ability in sexual selection.

## INTRODUCTION

Sexual selection, acting through female choice and male competition, is responsible for the evolution and maintenance of secondary sexual displays in males (Darwin 1871; Andersson 1994), such as bright colorations, calls, complex nests/arenas, and elaborated behaviours (Hill and Yasukawa 2014). Sexual signals often correlate with different (phenotypic and genetic) qualities of the bearers (Andersson and Simmons 2006) from which choosy females can gain direct and/or indirect benefits. Direct benefits of nonrandom mating include parental care, nuptial gifts, and access to resources that contribute to female's reproductive success (Hoelzer 1989; Meller 1994; Gubernick and Teferi 2000; Møller and Jennions 2001). Indirect benefits arise when offspring inherit the qualities of their father that enhance their viability (“good genes”: Hamilton and Zuk 1982; Milinski and Bakker 1990; Kirkpatrick 1996) and/or their mating potential (“sexy sons”: Fisher 1930; Weatherhead and Robertson 1979; Lande 1981; Borg 1982; Mead and Arnold 2004). However, only recent studies have started to explore the links between cognitive performances, secondary sexual traits and mating success (e.g. Boogert et al. 2008; Isden et al. 2013; Keagy et al. 2019; Rystrom et al. 2019).

Cognitive ability may aid in the development of complex sexual displays, leading to a positive association between the two. Indeed, in birds, song complexity was linked to the ability to solve different cognitive tasks (Boogert et al. 2008; Boogert et al. 2011a), suggesting that males can exhibit their cognitive ability through sexual signals (Searcy and Andersson 1986; Catchpole 1987, but see Sewall et al. 2013; Templeton et al. 2014; Anderson et al. 2017). In many species, males simultaneously display more than one sexual signal (Johnstone 1995). An extraordinary example is shown by bowerbirds, where males build and decorate a bower with sticks and brightly coloured objects and perform elaborated courtship displays to attract mates (Doucet and Montgomerie 2003; Keagy et al. 2009, 2011; Isden et al. 2013). This complex performance of different behaviours in concert requires high cognitive abilities and brain capacity (Madden 2001, but see Day et al. 2005). On the other hand, negative associations between secondary sexual traits and cognition may also arise by trade-offs because the development and maintenance of sexual signals are often costly (Emlen 2001; Allent and Levinton 2007). For example, a large number of sexually selected coloured ornaments in animals are based on dietary carotenoids (Griffith et al. 2006), but carotenoids also have other important functions, including the protection and development of neural structures (Johnson 2014; Erdman et al. 2015). Thus, the expression and maintenance of carotenoid-based ornament colouration may divert this resource away from neural and cognitive functions.

The male's cognitive ability may be subject to indirect sexual selection if it is either positively or negatively related to secondary sexual traits (Karino and Shinjo 2007; Boogert et al. 2011b; Madden et al. 2011). It is also possible that the male's cognitive ability is selected by females if it brings any direct or indirect benefits to females and the benefits outweigh the costs (Miller and Todd 1998; Hollis and Kawecki 2014). If cognitive abilities are positively correlated with other abilities in different contexts, such as foraging ability and predator avoidance, female selection on male cognitive traits may confer both direct and indirect benefits, thereby increasing offspring survival (Keagy et al. 2011; Rosenthal 2017). Although evidence of genetic effects on cognitive abilities is scarce in wild animals, some recent studies suggest that some cognitive traits, including inhibitory control, are heritable (Hopkins et al. 2014; Langley et al. 2020). Thus, females choosing mates with higher cognitive abilities may produce offspring with better cognitive abilities (Boogert et al. 2011b; Morand-Ferron et al. 2016). Thus, sexual selection may contribute to the evolution and maintenance of cognitive abilities in males (Hollis and Kawecki 2014). On the other side, this mate choice process may require females' ability to assess male cognitive ability or related sexual traits and result in the coevolution of both male and female cognition (Miller and Todd 1998; Candolin 2003; Keagy et al. 2009; Corral-López et al. 2017).

Our aims in this study were to investigate: 1) if male secondary sexual traits reflect their cognitive ability, 2) if female mate preference is affected by the interaction between female and male cognitive ability, and 3) if male attractiveness is determined by the interplay between secondary sexual traits and cognitive ability, by using three-spined stickleback (*Gasterosteus aculeatus*). Previous evidence shows that sticklebacks respond adequately in many cognitive domains, such as inhibitory control, and spatial and reversal learning, but with a great variation among individuals (e.g. Odling-Smee and Braithwaite 2003; Odling-Smee et al. 2008; Mamuneas et al. 2015; Rystrom et al. 2019 ; Bensky and Bell 2020). In this species, males defend a territory and provide intensive parental care by fanning the eggs and guarding the fry (Jakobsson et al. 1999) for which cognitive abilities may be important (Kotrschal et al. 2012; Samuk et al. 2014). A previous study showed that male cognition may be evaluated by prospective females in this species (Minter et al. 2017). However, it is still unclear how females assess male cognitive abilities and whether this assessment process is affected by their own cognitive abilities.

Male sticklebacks exhibit a series of courtship behaviours by first approaching a female while performing the “zig-zag dance,” “fanning” (i.e. intensive ventilating movements over his nest), and “gluing” (i.e. addition of glued kidney secretion to the nest). These complex behaviours may represent different aspects of male condition, for example, fanning indicating its ability to care for eggs (Östlund and Ahnesjö 1998) and gluing providing olfactory information of its reproductive state (Milinski et al. 2010). In addition, male courtship behaviours may potentially indicate its cognitive abilities (but see Minter et al. 2017). In this species, males build a complex nest with filamentous algae and kidney secretion not only to protect eggs (Wootton 1976) but also to attract mates (Barber et al. 2001; Östlund-Nilsson and Holmlund 2003; Head et al. 2017). The quality of a nest may reveal information about the builder's skills (Rushbrook et al. 2008) and cognitive performances (Schaedelin and Taborsky 2009). Male sticklebacks express a carotenoid-based red nuptial colouration on their cheeks and throat during the breeding season, and females preferentially mate with redder males (Östlund-Nilsson 2007), although the strength of this sexual selection varies among populations (Reimchen 1989) and could be affected by the honesty of the signal (Candolin 1999, 2000; Boughman 2007). Carotenoid-based coloration may be positively correlated with cognitive abilities if it mirrors their foraging skills (Mateos-Gonzalez et al. 2011), or negatively correlated if there is a trade-off between coloration and cognition mediated by carotenoid allocation (see Johnson 2014; Johnson 2014; Erdman et al. 2015; Erdman et al. 2015). We carried out our study in a stickleback population in which males express relatively weak red nuptial colouration (see Supplementary Figure S1; Supplementary Information), so we expect a minor influence of this trade-off on male mating strategies.

Here, we first evaluated the cognitive ability (i.e. inhibitory or self-control) of female and male sticklebacks in a detour-reaching task. In this task, fish needed to restrain behavioural propensity to obtain food reward. This inhibitory control is an indicator of the presence of complex cognitive processes (Kabadayi et al. 2018). We measured the performances of each individual fish during three consecutive days, thereby assessing both its initial ability to solve the task and its improvement over trials. Since individual personality may also affect the performance in the detour-reaching task (Rowe and Healy 2014), we additionally examined neophobic and exploratory behaviours in a novel environment and tested whether individual ability in the detour-reaching task was independent from their behaviour patterns. We then assessed nuptial colouration, courtship and nest construction of male sticklebacks. We expected that males with better courtship performances, more elaborate nests, or redder throats would have higher scores in the detour-reaching task. In a dichotomous-choice test, we evaluated female preference by exposing each female to two different males with contrasting performance (relatively good and bad) in the detour-reaching task. We expected that females, especially those with high cognitive abilities, would choose males showing better performance in the detour-reaching task. Finally, by using structural equation models, we evaluated the direct and indirect (through secondary sexual traits) causal relationships between the detour-reaching task score and male attractiveness (assessed by the repeated female preference tests).

## MATERIAL AND METHODS

### Study system and holding conditions

A total of 49 juvenile three-spined sticklebacks were captured in the Rio Miñor (Galicia, Spain) in early November 2018. In this annual population, male colouration during the breeding season is not prominent in comparison to other adjacent stickleback populations (Supplementary Figure S1). The fish were housed randomly in five outdoor holding tanks (filled with 260 L water), each housing 9 or 10 fish and containing a filter and a shelter made of ceramic hollow brick and a roof tile. Fish were fed three times per week on a commercial pelleted diet (Gemma Micro, Skretting, Norway), containing a high level of carotenoids (103.9 μg g^−1^, Kim and Velando 2016). At the beginning of the breeding season (in March), the fish were moved to indoor aquaria systems, where they were individually housed in 8-L tanks. The tanks were connected to closed flow-through water systems in which water was continuously filtered, aerated and temperature-controlled. The lateral walls of the tanks were opaque, so preventing visual contact between individual fish. The natural photoperiod and water temperature were simulated by programmed illumination and a water-cooling system (light: dark 12 h:12 h and 13 °C in March). The fish were fed daily on moistened food pellets, which sink on the bottom of the tank, to habituate them to bottom-feeding for the detour-reaching task (see below).

In early April, we provided males (*N* = 22) with a Petri dish filled with 90 g sand and one hundred 5 cm green polyester threads. Most male sticklebacks from our study population readily use polyester threads, which mimic naturally available nest-building materials, and typically build a nest on the sand. We presented a gravid female enclosed in transparent glass to each male for 5 min twice a week during 9 weeks until early June to prompt nest construction behaviour and the maintenance of nuptial colouration. Each female was presented to all the males across different days, so all females and males were shown to each other at least once. Courtship behaviours of males were video recorded (see below) during the last presentation of a gravid female until which all males completed their nests (evident from the presence of the nest entrance).

### Cognitive ability test, detour-reaching task and exploration behaviour

In late May, when the males had started (*N* = 10) or finished nest construction (*N* = 12), the cognitive performance of all survived fish (*N* = 22 males and 20 females) was evaluated in a simple detour-reaching task in which the fish needed to find the entrance to reach the food. Detour tasks using transparent barriers are commonly used in animals, including fish, to test individual ability to access a reward that can be seen but is not directly accessible, and provide strong predictive measures of inhibitory control skills (Vlamings et al. 2010; MacLean et al. 2013; Lucon-Xiccato et al. 2020). Thus, fish needed to inhibit the impulse to reach for the food directly, bumping into the transparent barrier, to successfully retrieve the reward (MacLean et al. 2014; Minter et al. 2017; Kabadayi et al. 2018). Inhibitory control is considered especially important for behavioural flexibility (Manrique et al. 2013), hence it probably influences courtship and parental care of male sticklebacks (Keagy et al. 2019). Inhibitory control abilities are also crucial for decision-making (Coutlee and Huettel 2012), probably including mate choice decision.

The apparatus used here consisted of a transparent plastic cup with a circular entrance (diameter 3 cm) outlined in blue colour and located on the top of the same individual tank in which each fish was hold (Supplementary Figure S2). In this test, fish should find the entrance to access a reward inside the cup, which is visible from all directions through the transparent wall of the apparatus. Before the test, the fish were exposed to the apparatus, which was placed in their respective individual tanks (without food rewards) for 24 h, to avoid neophobia and for behavioural adaptation (see also Álvarez-Quintero et al. 2020). In the trial after the 24 h exposure, the focal fish was familiar with the apparatus, showing no indications of neophobia, and tried to access the apparatus once the food (moistened pellets) was provided inside the apparatus. When the trail began, fish typically swam directly to the transparent bottom of the cup, where the food was deposited, and repeatedly tried to reach the food. In order to retrieve the food, the fish needed to swim about and into the cup through the outlined opening. Fish's performance in the trial was observed in situ up to 3 h to measure the time taken for the fish to enter the apparatus through the outlined entrance. Once the trial ended, we removed the apparatus from the tank and the remaining food (if not eaten during the trial) was left in the tank. In the following two days, the same tests were performed immediately after the apparatus was introduced into the tank and food provided inside the apparatus (hereafter second and third trials). We assigned the maximum time (180 min) if a fish did not successfully enter the apparatus (first trial: 13 out of 42 fish; second trial: 12 fish; third trial: 7 fish). A decrease in the time to enter the apparatus over the three repeated trials may indicate learning of inhibition control, which was facilitated by the outlined entrance (Tommasi et al. 2012).

Cognitive ability may be also affected by noncognitive factors (Rowe and Healy 2014; Griffin et al. 2015), such as exploration behaviour and neophobia (see Bensky and Bell 2020). Thus, we additionally evaluated individual behaviours in a novel environment in an independent behavioural assay in order to test whether the detour-reaching task score of an individual represents its cognitive ability irrespectively of its behaviour patterns. Nine days after the detour-reaching task, exploratory and neophobic behaviours of individual fish were tested in an observation tank (25 cm × 15 cm, 7 cm water depth). The tank contained two easily accessible open compartments, each including a coloured Petri dish with the same amount of food (diameter 5.5 cm; blue or green; Supplementary Figure S3). A tripod-mounted digital video camera (Sony; Handycam HDR-CX405) was located above the tank for video recording of fish behaviours without disturbance. For each test, a focal fish was carefully netted and moved from its holding tank to the observation tank and held inside a transparent cylinder (9 cm diameter) at the opposite end from the inner compartments (Supplementary Figure S3) during the acclimatization period. After 30 s of acclimatization, the fish was released from the cylinder and allowed to swim freely for 300 s. We analysed the video to measure the time taken for the fish to approach any of the two Petri dishes containing food and the total time spent moving during the test as proxies of neophobia and exploratory activity, respectively.

### Courtship behaviours and nest size

Male courtship behaviours were video recorded by using a digital camera (Sony; Handycam DCR-SX44) during the last presentation of a female in each male (in early June, see above). By analysing the 5-min videos, we quantified different courtship behaviours following Minter et al. (2017). We counted 1) the number of zigzag dance toward the female, 2) the number of leads, that is when the male swims back to the nest, 3) the number of fanning events, 4) the number of gluings, and 5) the number of entrance showings, that is when the male puts his head into the nest showing the entrance. The number of each behaviour per minute was used for data analysis.

In the peak breeding season (early June), each male's nest (*N* = 22) was photographed along with a scale for calibration by using a digital camera (Nikon D90, Nikon Corp., Tokyo, Japan) from above the males' tank. Then, following Barber et al. (2001), we measured the size of the nests from the digital images by using the ImageJ software (Rasband 1997). We estimated the nest's total area, as the polygon enclosing all visible nest material, and the bulk area, as the surface of the nest through which no basal substratum was visible (i.e. completely covered by nest material). Additionally, we scored the use of material for nest construction (hereafter “nest material”) ranging from 0 to 5 based on the proportion of threads used for nest construction with respect to the total number of threads provided (a total of 100; ≤10% score 0, 11–25% score 1, 26–50% score 2, 51–75% score 3, 76–90% score 4 and 91–100% score 5).

### Female preference test

During June, female mate preference was assessed in a commonly used dichotomous-choice test (see Candolin 1999) in which a gravid female was exposed to two males with contrasting performance (relatively good and bad) in the detour-reaching task (*N* = 19 females). One female was excluded from the study because it never became gravid. Males were categorised according to the individual mean of three consecutive detour-reaching task outcomes (i.e. time taken to enter the apparatus) as good performance individuals (below the mean: 77.39, *N* = 12; range 1.33–63.67) and bad performance individuals (above the mean, *N* = 10; range 83.33–180). In each preference test, a pair composed by a good and a bad male, which were clearly distinguished by their detour-reaching task performance (mean difference ± SE: 130.71 ± 27.01 min), were used.

The dichotomous-choice arena consisted of a rectangular tank (50 cm × 30 cm, 14 cm water depth), which contained a focal female moved from its holding tank (hereafter female tank), and two male tanks (25 cm × 15 cm, 14 cm water depth). The same holding tanks in which the two focal males were individually housed and built a nest were used as male tanks in the preference test to avoid stress caused by any manipulation and allow the males to court normally (Ramsay et al. 2009). The walls of the tanks were opaque except those that allowed visual contact between the focal female and each male. The two males were visually isolated from each other and had the same lighting conditions. Lines were drawn on the bottom of the female tank to distinguish different zones: a right preference zone adjacent to the right male tank, a left preference zone adjacent to the left male tank, and a no-preference zone 20 cm away from the male tanks (Supplementary Figure S4). A tripod-mounted digital video camera (Sony; Handycam DCR-SX44) was located over the experimental set up for video recording of the preference tests.

Before each preference test, a fully gravid female (evident from its abdomen size and the dilatation of its genital opening) was carefully netted from its tank and placed inside the female tank, the two male tanks were moved and positioned, and then the fish were acclimatized for 5 min, during which visual interaction between them was blocked by a removable opaque plastic divider. Then, the female was enclosed inside a transparent plastic cylinder (9 cm diameter) and positioned in the middle of the no-preference zone, and the plastic divider between the female and the males was removed to allow visual inspection and stimulation for 1 min, after which the cylinder was also removed. The test began when the cylinder was removed and lasted for 10 min. The time that the female spent in each of the two preference zones (right and left preference zone) was determined by video analysis. Female preference for each male was calculated as the time spent in each preference zone divided by the total time spent in the two preference zones. Thus, the female preference for a male ranged from 0 to 1, where 1 indicates an absolute preference, 0.5 indicates no preference, and 0 indicates no absolute preference (i.e. an absolute preference for the other male). The total time spent in the preference zones was not related to the female's detour-reaching task score (Pearson's correlation test: *R* = −0.20, *P* = 0.139).

We repeatedly assessed mate preference of each gravid female in two consecutive tests with different pairs of males on the same day. The second test started aproximately 10 min after the first test ended. The position (right or left tank) of the males of different categories (good and bad detour-reaching task performances) was alternated between the two consecutive tests to control for any possible lateral preference of females (Bisazza and Brown 2011). Eight out of 19 females became fully gravid again (i.e. second gravidity during the test period; hereinafter gravidity event) after the first two tests within the study period and their mate preference was re-evaluated in two more consecutive tests. Thus, we conducted a total of 56 tests, and each female was tested 2 or 4 times. Each male was repeatedly used in different preference tests (range 3–8 times, mean ± SE: 4.9 ± 1.2 times). Bad-performing males were used slightly more times than good-performing males, but this difference was not significant (GLM: LRT, $\chi_{1}^{2}$ = 3.289, *P* = 0.069). Each male was used only once in a day. All pairs of males were different across the 56 tests.

### Fish size and nuptial colour

Once the preference tests finished (late June), the standard length of all study fish was measured (to the nearest 1 mm). In addition, males were photographed on their lateral side by using a digital camera (Nikon Corp., Nikon D90) under standardized conditions. We then calculated the relative size of the red nuptial colour area in relation to the total lateral body area from the digital images by using image analysis software (Olympus, analySIS FIVE) and following a previously described protocol (Kim et al. 2016).

### Statistical analyses

All analyses were performed using the R Statistical Package (R Core Team, 2018, version v.3.5.2), and *P*-values are based on two-tailed tests.

### Detour-reaching task scores

Detour-reaching task outcomes (i.e. time taken for a fish to enter the apparatus) were transformed using GuanRank, a nonparametric ranking-based technique that converts right-censored data into a linear space of hazard ranks (Huang et al. 2017). A higher guanrank-transformed value indicates a shorter time to pass the entrance and thus a stronger ability to solve the detour-reaching task. These guanrank-transformed detour-reaching task scores from the three repeated trials were analysed in a linear mixed model (LMM) by using the *lme4* package (Bates et al. 2014). In the model, we included trial day (1, 2, and 3), sex and standard length as fixed effects, and the individual identity (intercept) and the individual change across trials (slope) as random terms (i.e. random regression). The significance was determined by *F* test with Satterthwaite approximation for degrees of freedom using the *lmerTest* package (Kuznetsova et al. 2017). Predicted values were plotted using the *sjPlot* package (Lüdecke 2018). We also assessed the within-individual repeatability of detour-reaching task scores using the *rpt* function in the *rptR* package (Nakagawa and Schielzeth 2010).

### Relationships between detour-reaching task score and secondary sexual traits in males

A principal component analysis (PCA) was performed on z-transformed courtship behaviours (i.e. the numbers per minute of zigzag dance, leads, fannings, gluings, and entrance showings) using the *prcomp* function of the *stats* package (R Core Team 2013). These different courtship behaviours were strongly correlated with each other (Supplementary Table S1). The first two components of the PCA account for more than 85% of the overall variation (Table 1). The first component (PC1) correlated with all courtship behaviours, and the second component (PC2) correlated positively with the number of leads and entrance showings, but negatively with the number of gluings, fanings, and zig-zags (Table 1).

**Table 1:** Loadings of the PCA of courtship behaviours. We used the PC1 in the analyses that assess the relationship between detour-reaching task score and courtship

Component	PC1 eigenvector	PC2 eigenvector
Eigenvalue	3.656	0.609
Variance (%)	73.125	12.180
Cumulative variance	73.125	85.306
Variable		
N of zigzags	0.922	−0.158
N of leads	0.750	0.606
N of fanning events	0.929	−0.023
N gluings	0.848	−0.450
N of entrance showings	0.813	0.116

We analyzed how the individual average detour-reaching task score (guanrank-transformed) of males was related to their secondary sexual traits, nest characteristics (total area, bulk area, and index of the amount of nest material), courtship behaviours (PC1_courtship_ and PC2_courtship_) and nuptial colour (relative red area), in separate linear models (LMs), each including one sexual trait as an independent variable. Before analysis, the nest bulk area was log-transformed to improve data distribution. Effect sizes for the relationships between male traits and detour-reaching task score in the linear models were estimated using the *effectsize* package (Ben-Shachar et al. 2020).

### Female preference

Female preference for each male (i.e. the proportion of time spent by a female in the corresponding preference zone) was analyzed in a generalized linear mixed model (GLMM) with beta-binomial error distribution (Crawley 2012; Harrison 2015), using the *glmmTMB* package (Brooks et al. 2017). The model included the male detour-reaching task performance (MDTP) (good or bad), the female detour-reaching task score (FDTS) (guanrank-transformed), the female gravidity event (i.e. first and second gravidity during the test period), and the male position (right or left) as fixed terms. We also included the following two-way interactions: MDTP × female gravidity event and MDTP × FDTS. Male identity, and test identity (i.e. each preference test) nested within female identity were included as random terms. Predicted slopes and confidence intervals were plotted by using *visreg* package (Breheny and Burchett 2017). The significance of terms was determined by the Likelihood Ratio Test (LRT), and post-hoc comparisons were performed using Tukey's post hoc test by using the *TukeyHSD* function. Effect sizes for explanatory variables were calculated using the *effectsize* package (Ben-Shachar et al. 2020).

### Structural equation modelling on male attractiveness

We used structural equation models (SEMs; see Shipley 2009) to examine direct and indirect causal links between detour-reaching task score and male attractiveness by using the *psem* function of the *PiecewiseSEM* package (Lefcheck 2016). The *PiecewiseSEM* package offers a flexible mathematical framework, which relaxes some important limitations of standard structural equation models, such as the requirement of a large sample size (Lefcheck 2016). As a measure of attractiveness of each male, we used the average proportion of time engaged with females (i.e. time spent by females in the corresponding preference zone) in all preference tests in which the particular male participated. This average was not related to the number of times that the male was involved in the preference tests (*N* = 22; *R* = −0.27, *P* = 0.22). The average was z-score transformed, controlling for the male's position in the choice arena (right or left tank), by using *scale_by* function in the *standardize* package (Eager 2017). We explored both the direct link between male detour-reaching task score and male attractiveness and the indirect paths through the links with the secondary sexual traits (nest bulk area, courtship behaviours [PC1_courtship_ and PC2_courtship_], and relative red area). We omitted the directionality of the path between detour-reaching task score and relative red area because both variables could act as a predictor or response variable due to possible trade-offs. All variables were z-score transformed (i.e. a mean of zero and a standard deviation of one).

We applied directed separation to the set of independent claims, following a direct acyclic graph. In each step, the *piecewiseSEM* also tests the assumption that there are no missing or incomplete relationships among unconnected variables (Shipley 2013). We selected the final model using Shipley's extension for the Akaike Information Criteria (AIC; Shipley 2013), and evaluated its goodness of fit using the Fisher's *C* statistic (i.e. test of directed separation; Shipley 2009). This statistic can be compared with a χ ^2^-distribution with *k* × 2 degrees of freedom, where *k* is the total number of independence claims specified in the model; *P* > 0.05 indicating that the model adequately reproduces the hypothesized causal relationships (*P* > 0.05; Shipley 2009; Lefcheck 2016).

## RESULTS

### Detour-reaching task scores

**Figure 1:**
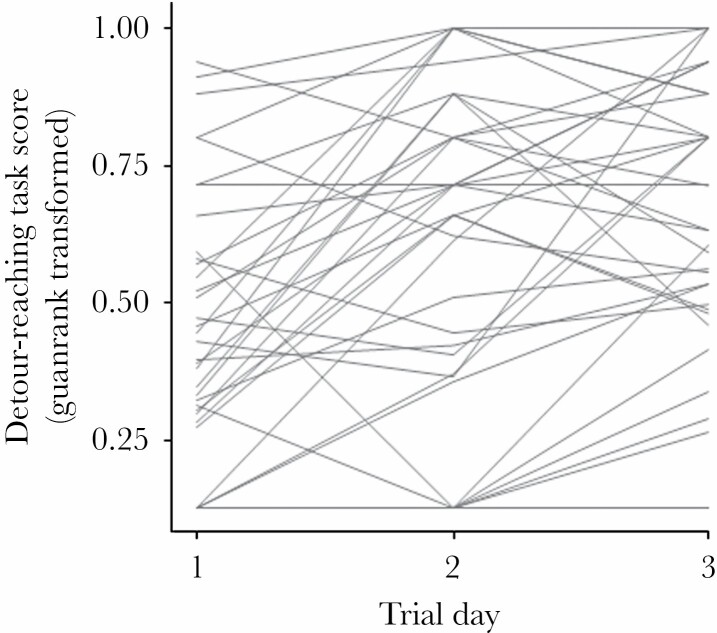
Changes in the detour-reaching task scores over repeated trials, expressed as the guanrank-transformed time taken to reach the food (higher values indicating a shorter time to solve the task), in three-spined stickleback (*N* = 42). Lines represent the performance of different individuals across trials.

Fish improved their performance in the detour-reaching task throughout the repeated trials (LMM, *F*_1, 41_ = 25.94, *P* < 0.001; Figure 1). Males and females did not differ in their ability to solve the task (LMM, *F*_1, 39_ = 0.88, *P* = 0.354), which was not related to individuals' body size (i.e. standard length; LMM, *F*_1, 39_ = 0.15, *P* = 0.699). Importantly, the task scores were highly repeatable across trials within individuals (*N* = 42 individuals × 3 trials; *R* ± SE = 0.64 ± 0.07, *P* < 0.001), and random intercepts and slopes were correlated (*N* = 42; Pearson's correlation test: *R* = 0.59, *P* < 0.001), suggesting that faster problem-solvers were also better learners (Figure 1). The individual mean of three consecutive detour-reaching task scores was strongly correlated with both the individual intercept (*N* = 42; *R* = −0.88, *P* < 0.001) and slope (*N* = 42; *R* = −0.71, *P* < 0.001) estimated from the random regression model, suggesting that this average value is a good synoptic descriptor of individual performance.

On the other hand, the mean detour-reaching task score of fish did not correlate with their neophobic behaviour measured in an independent assay (time to approach a novel object; *N* = 42; *R* = 0.083, *P* = 0.599) and exploratory activity (total time moving; *N* = 42; *R* = 0.020, *P* = 0.901) observed in a novel environment.

### Male detour-reaching task scores and sexual traits

**Figure 2:**
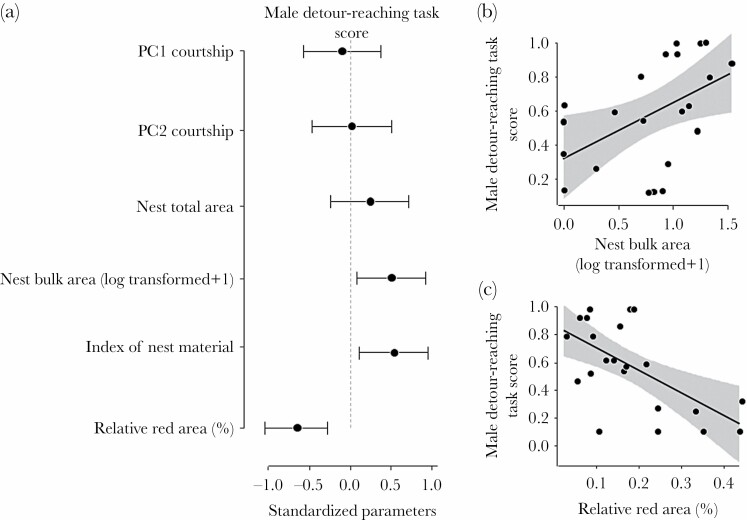
(a) Standardized coefficients and 95% CIs for male traits in the linear models assessing the relationships between male traits and reaching-task score. Relationships between detour-reaching task scores and (b) the relative red area and (c) bulk area of the nest (log-transformed +1) (*N* = 22 males). A higher value of the detour-reaching task score indicates a shorter time to solve the task.

Neither courtship performance nor the total area of the nest were related to the detour-reaching task score (LMs; PC1_courtship_, *F*_1, 20_ = 0.25, *P* = 0.620; PC2_courtship_, *F*_1, 20_ = 0.002, *P* = 0.968; nest's total area, *F*_1, 20_ = 1.19, *P* = 0.289, Figure 2a). Males that built nests with larger bulk areas and higher values in the index of nest material had higher detour-reaching task scores (LMs; bulk area: *F*_1, 20_ = 6.39, *P* = 0.020; nest material: *F*_1, 20_ = 7.51, *P* = 0.013, Figure 2a,b). Male's nuptial colour was negatively related to the ability to solve the detour-reaching task (LM, *F*_1, 20_ = 12.37, *P* = 0.002; Figure 2a). Males with larger red areas showed significantly lower detour-reaching task scores (Figure 2c).

### Female mate preferences

**Figure 3:**
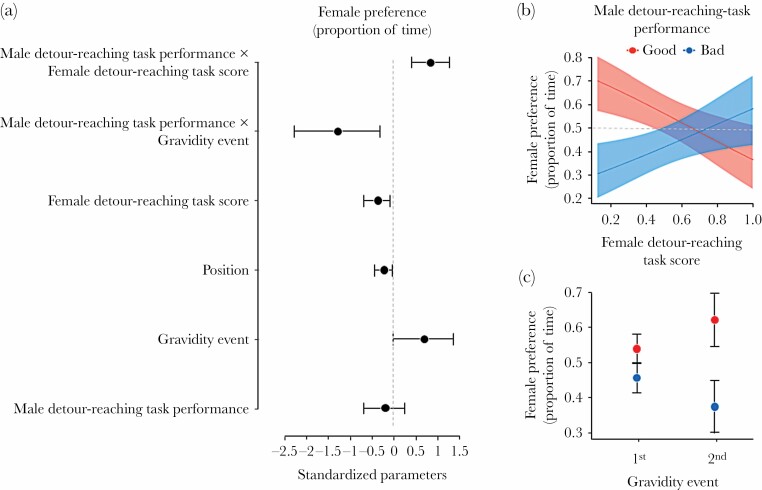
(a) Standardized coefficients and 95% CIs for independent variables in the analysis of female preference. (b) Relationship between detour-reaching task score of females and their preference (i.e. proportion of time spent) for males with contrasting performances in the detour-reaching task (good: *N* = 12; bad: *N* = 10); the dashed line indicates no preference for any male. A higher value of the detour-reaching task score indicates a shorter time to solve the task. (c) Differences in female preference (mean proportion of time ± SEM) for males with contrasting performances according to the gravidity event (1st and 2nd gravidity during the test period).

In the dichotomous-choice test, female preference was affected by the interaction between the MDTP (good or bad) and the female gravidity event (first and second gravidity during the test period) (Table 2). On the second gravidity, the females spent more time interacting with “good” males than “bad” males (Tukey post-hoc test, *P* = 0.03), while on the first gravidity, although “good” males also tended to be preferred, the difference was not significant (Tukey post-hoc test, *P* = 0.15). The female preference was also affected by the interaction between the MDTP and the FDTS (Table 2). This is because females with the highest scores showed no preference, whereas the others showed a strong preference for the males with good detour-reaching task performances (Figure 3b). We also found that females preferred to interact with males located in the left zone (Table 2).

**Table 2:** Summary of the GLMM analysis of female mate preference. Significant P-values are highlighted in bold

	Mate preference		
Variable	Estimate ± SE	$\chi_{1}^{2}$	*P*
Intercept	1.79 ± 0.47		
MDTP [bad]	−2.03 ± 0.50	7.28	**0.007**
FDTS	−1.48 ± 0.56	0.00	1.00
Gravidity event (second gravidity)	0.68 ± 0.36	0.00	1.00
Male's spatial position	−0.51 ± 0.21	6.24	**0.012**
MDTP × FDTS	2.97 ± 0.80	13.88	**<0.001**
MDTP × gravidity event	−1.36 ± 0.51	7.03	**0.008**

### Structural equation modelling

**Figure 4:**
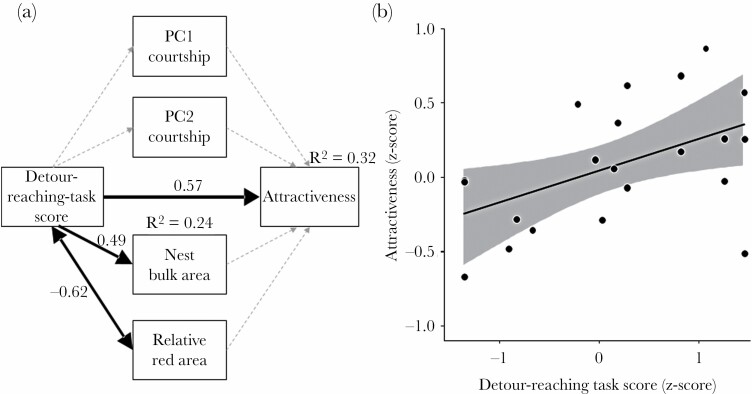
(a) Structural equation model testing direct and indirect paths between attractiveness (i.e. the average time the females spent with a focal male in different tests) and problem-solving ability (detour-reaching task score) of males. Indirect paths included courtship (PCs), nest structure (bulk area), and relative red area. The relationship between detour-reaching task score and relative red area was modelled as a correlation (i.e. no causal link between them) and represented by a double-headed arrow. The grey dotted arrows represent the paths tested in the saturated model but not included in the final model (see Results) of which paths are indicated by black solid arrows, and numbers are standardized effects. Arrow widths are proportional to the magnitude of the standardized regression coefficients. (b) Relationship between attractiveness and detour-reaching task score of males (*N* = 22).

The final SEM (with the lowest AIC; see Figure 4) was supported by the data (*C* = 2.77, *P* = 0.378; Table S2), suggesting no significant missing paths in this model (Lefcheck 2016). Males with high scores in the detour-reaching task were more attractive to females (Figure 4b). Thus, the detour-reaching task score had a direct positive effect on male attractiveness (path weight ± SE = 0.57 ± 0.09, *P* = 0.006; Figure 4), but no indirect effects via sexual traits were detected. Thus, the direct effect of detour-reaching task score explained 32% of the variation of male attractiveness.

The detour-reaching task score had a direct positive effect on the nest bulk area (path weight ± SE = 0.49 ± 0.20, *P* = 0.020). The detour-reaching task score and the relative red area were negatively correlated (*R* = −0.62, *P* = 0.002). However, these secondary sexual traits did not influence male attractiveness (Table S2; Figure 4a), indicating no indirect link between the male detour-reaching task score and attractiveness through the sexual traits.

## DISCUSSION

Our results show that individual performance in the detour-reaching apparatus was highly repeatable, and individuals that showed good performance in the initial trial were also good learners, improving their performance more rapidly than bad-performing males. The males' ability to solve the detour-reaching task was mirrored in the nest structure (bulk area and nest material), probably because good cognitive skills are required to build a high-quality nest (Schaedelin and Taborsky 2009; Hall et al. 2015; Edwards et al. 2020). Contrary to our prediction, females with the strongest ability to solve the detour-reaching task did not show any preference between potential mates with different performance in the detour-reaching task, whereas the other females preferred males with good detour-reaching task performance. Overall, male attractiveness to females was directly and positively related to the male's detour-reaching task score, but there was no evidence that females use secondary sexual traits of males (courtship, nest structure, and red colouration) to evaluate their cognitive ability. Since females did not observe male's detour-reaching task performance prior to the preference test (see Chen et al. 2019 for a direct preference for problem solvers) (but see Camacho-Alpízar et al. 2020), they might discriminate males by other characteristics correlated to cognitive ability than those measured in this study. The detour-reaching task score of males was negatively correlated with their nuptial colour, suggesting a possible trade-off between ornament expression and cognitive functions.

In this study, we evaluated cognitive ability of fish in a test where they can observe food from all directions but need to find the entrance to access the reward. This test measured their ability to solve a problem by inhibiting automatic responses (direct swimming to food) and flexibly adjust behaviours (Diamond 1990; Santos et al. 1999; Vlamings et al. 2010; MacLean et al. 2013). The exploratory and neophobic behaviours of individuals were assessed in a novel environment (a different behavioural assay from the detour-reaching task), but they were not related to the time taken to access the food in the detour-reaching task. This result suggests a minor influence of these personality traits on the ability to solve this task, probably because fish were familiarised with the detour apparatus before the test. In sticklebacks, it has been shown that decision-making accuracy is similar between bold and shy individuals, although shyer fish make slower decisions (Mamuneas et al. 2015).

Remarkably, individual fish in our study showed consistent but improving solving abilities across repeated detour-reaching task trials (i.e. learning), and their initial performance (intercept) was correlated to the degree of improvement (slope). Thus, the inhibitory control was repeatable within individuals and related to their learning ability, which reflect their cognitive skills (see Morand-Ferron et al. 2016; Camacho-Alpízar et al. 2020). The detour-reaching task scores were also correlated to the nest structure, which possibly represents the builder's cognitive abilities, such as planning and manipulative capacities (Bshary et al. 2002; Hansell and Ruxton 2008). Thus, our results suggest that the detour-reaching task performance represents some particular aspects of cognitive abilities. Nevertheless, we acknowledge that further studies, using different cognitive assays, are required to assess whether our results on the male and female cognitive abilities may be understood within the more general scope of cognition (Rowe and Healy 2014).

We expected that females with better cognitive abilities, and presumably with better discriminatory skills, would be more selective (Corral-López et al. 2017). We speculate that inhibitory control may be relevant to mate choice if “self-controlled” females are able to postpone their mating decisions until they encounter the most appropriate male. However, females with medium-low scores in the detour-reaching task, but not those with the highest scores, preferred males with better cognitive abilities in the preference test. Previous evidence suggests that female sticklebacks with better cognitive skills spend more time evaluating potential mates (Rystrom et al. 2019). The differences among females in their preference (measured as time interacting with a particular male) for more skilled males might arise if, for example, females with higher cognitive abilities spent equal time evaluating both males for later decision-making. We assessed female preference in a widely used dichotomous test (e.g. in sticklebacks; Bakker et al. 1999; Pike et al. 2007; Rystrom et al. 2019) in which physical interactions between the focal female and candidate males were prevented. These interactions may be important, especially for those females that need more elements to evaluate a potential mate. It is also possible that females with lower cognitive abilities showed a stronger preference due to increased potential benefits when mating with more skilled males (Cotton et al. 2006; Holveck and Riebel 2010), but we have no data to examine this possibility. Although the mechanism is unclear, our study suggests that female mating preference differs according to their own cognitive abilities. This differential mate preference may produce disassortative mating for cognition, which would affect the evolution and maintenance of both male and female cognitive ability.

The female preference for males with better cognitive abilities was evident especially in the latter two preference tests (in the second gravidity during the test period). This may suggest that females changed their preference according to the past social-sexual interactions (Brown et al. 2011), becoming more selective after the exposure to multiple males (Jennions and Petrie 1997; Walling et al. 2008, Fowler-Finn and Rodríguez 2012). Interestingly, we also found that females preferred to interact with males located in the left zone. Thus, the spatial location of nest may have important consequences for male mating success (see Bolnick et al. 2015).

In some species, cognitive performance covaries with fitness-related traits, such as reproductive success and survival (e.g. Keagy et al. 2009, 2011; Cole et al. 2012; Cauchard et al. 2013; Ashton et al. 2018), and hence nonrandom mating based on the partner's cognitive ability may bring direct or indirect benefits to females. Our structural equation model analysis revealed that males with better cognitive abilities were also more successful in attracting the female's attention. Our results confirm previous evidence from a study showing that female sticklebacks were more likely to enter into the nest of males with higher learning abilities in no-choice mating trials (Minter et al. 2017). Since females did not directly observe the males performed in the detour-reaching task, they had to indirectly evaluate them by other correlated traits displayed during the courtship encounters. In accordance with Minter et al. (2017), we did not find any evidence supporting that the studied sexual traits indirectly mediate the relationship between male attractiveness and the ability to solve the detour task.

The nest structure and quality mirrored the cognitive ability of builders, probably because the nest-building activity requires cognitive skills and brain functions to coordinate complex behavioural processes (see Bshary et al. 2002 and references therein). However, this link between cognitive ability and nest structure was not involved in the attractiveness of better cognitive-skilled males in our study. In the preference test, females could observe the male's nest, but from a distance, because the tank walls prevented them from swimming over the nests. Thus, we cannot discard the possibility that, in natural conditions, nest structure may be evaluated by prospective females and influence their mate choice. On the other hand, the male's ability to solve the detour-reaching task was negatively correlated with carotenoid-based red colouration, suggesting a possible trade-off between this sexual signal and cognitive performance. Dietary carotenoids are required for a variety of cognitive and motor functions (e.g. Larcombe et al. 2008; Johnson 2014; Christensen et al. 2020), so trade-offs may arise due to carotenoids allocation between these functions and their use as skin pigments, especially when dietary carotenoids are scarce (Catoni et al. 2008). In our study, the male traits used by females to evaluate cognitive ability of potential mates remain unrevealed. Nevertheless, it is possible that female sticklebacks simultaneously assess multiple traits for decision-making (Johnstone 1995; Miller and Todd 1998; Künzler and Bakker 2001; Candolin 2003).

Sexual selection based on cognitive ability in this population may give rise to the (co)evolution of secondary sexual traits in different directions. Although selection for red colouration may be also affected by male competition and predation, it is known as an important criterion for female choice in many stickleback populations (reviewed in Rowland 1994). However, we did not find any evidence of female preference for males with large red area in our study population. It is interesting to note that males in this population express relatively weak red coloration on their throat. In this annual population, sticklebacks breed with extreme frequency during a single breeding season like in other nearby populations (Kim et al. 2017), and it is likely that successful males simultaneously take care of multiple clutches from several females in their nests. Thus, mating with males that build large and solid nests may bring both direct and indirect fitness benefits to females. Selection on male cognitive ability and female preference might drive the evolutionary loss of intense sexual colouration, which is negatively correlated with male cognitive ability. Further studies should explore the mechanisms underlying female mate choice for cognitive ability and the role of the trade-off between male colouration and cognitive ability in the evolution of male ornamentation.

## FUNDING

This work was supported by the Spanish Ministerio de Ciencia, Innovación y Universidades (PGC2018-095412-B-I00 and RYC-2015–18317) and the Consellería de Cultura, Educación e Ordenación Universitaria, Xunta de Galicia (ED431F 2017/07). N.A.Q. was supported by FPI student grant from the Ministerio de Ciencia, Innovación y Universidades (BES-2016–078894). Funding for open access charge: Universidade de Vigo/CISUG.

We would like to thank Jose C. Noguera and Belén Otero for their help during the study and J. Keagy and an anonymous reviewer for their constructive comments on an earlier version of the manuscript.

Conflict of interest: The authors declare no conflict of interest.

Ethical considerations: This experiment was approved by the Animal Experiment Ethics Committee of the Universidade de Vigo and the Xunta de Galicia (ES360570181401/19/FUN01/BIOL AN.08/SYK).

Data accessibility: Analyses reported in this article can be reproduced using the data provided by Álvarez-Quintero et al. (2021).

## Supplementary Material

arab052_suppl_Supplementary_Material
