# Supplementary material for: Smart mating: the cognitive ability of females influences their preference for male cognitive ability
Source: Behav Ecol. 2021 Jun 22;32(5):803–13. doi: 10.1093/beheco/arab052 (PMC8528552; doi:10.1093/beheco/arab052)
Supplement: arab052_suppl_Supplementary_Material [file arab052_suppl_supplementary_material.docx]

**SUPPLEMENTARY INFORMATION (SI)**

**SUPPLEMENTARY FIGURES**

**Figure S1.** (a) Variation in nuptial coloration of mature male sticklebacks at the study population. A nuptial coloration gradient is represented from duller (1) to redder (4) individuals and (b) a male stickleback from another nearby population showing a prominent nuptial colouration.


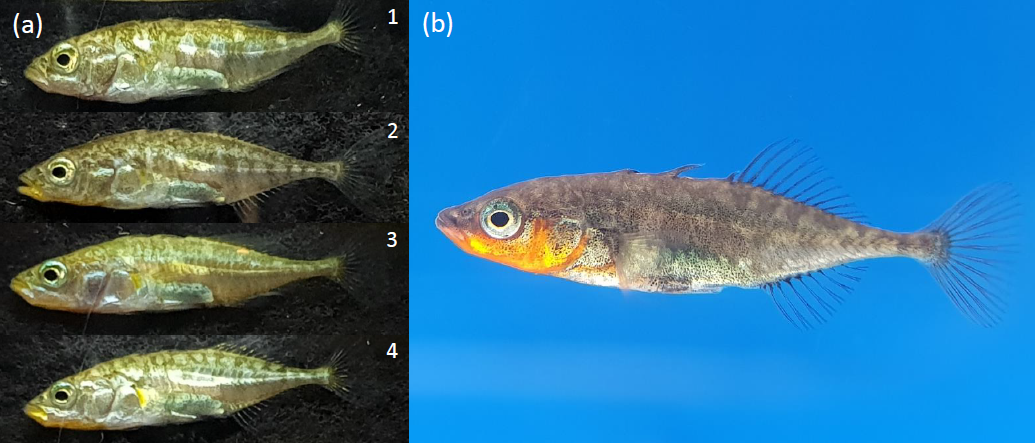


**Figure S2.** Apparatus used to assess the detour-reaching task, a transparent plastic cup with a blue-highlighted entrance, which was placed on the top of the individual tank. Food reward was given on the bottom of the apparatus at the onset of the test.

-
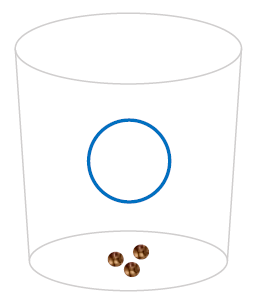


**Figure S3.** The observation tank used to assess the neophobic and exploratory behaviours of the experimental fish. The exterior of the tank was opaque to prevent external influences and the inner compartments were divided by a transparent wall. Fish was hold inside a cylinder prior to the test (circled position). The water temperature in the observation tank was the same as in the home tanks.


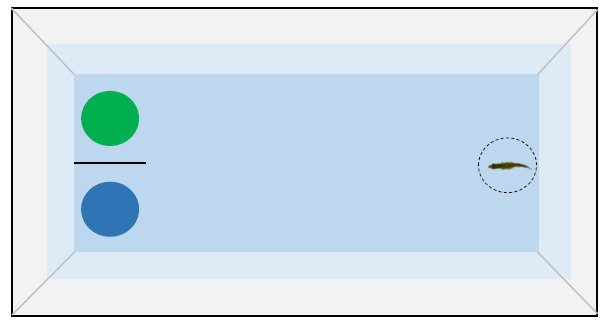


**Figure S4.** Design of the dichotomous choice test used to assess the mate preference of females. The female and male symbols indicate the location of the female and males in the experimental setup. The circle indicates the position of the female (hold inside a cylinder) prior to the test. Dashed lines on the bottom of the female tank delimited a left preference zone; adjacent to the left male tank, a right preference zone; adjacent to the right male tank, and a no-preference zone. The water temperature in the test tank was the same as in the home tank.


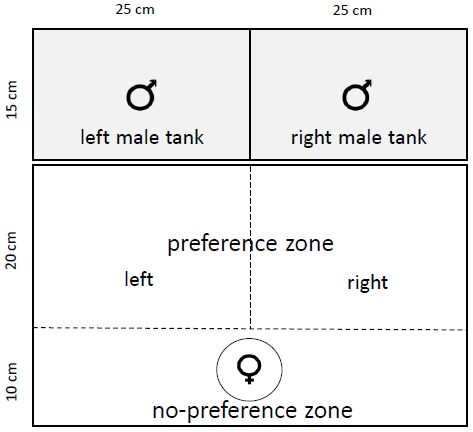


**SUPPLEMENTARY TABLES**

**Table S1.** Correlations between the five measures of courtship (number of zig-zags, leads, fanning events, gluings, and entrance showings, all transformed to z-score). These different courtship behaviours were highly correlated to each other.

| ***N* = 22** | **N of zig-zags** | **N of leads** | **N of fanning events** | | **N of gluings** | **N of entrance showings** |
| --- | --- | --- | --- | --- | --- | --- |
| **N of zigzags** | -- | *R* = 0.61  *P* = 0.002 | | *R* = 0.82  *P* = 0.000 | *R* = 0.82  *P* = 0.000 | *R* = 0.66  *P* = 0.001 |
| **N of leads** | *R* = 0.61  *P* = 0.02 | -- | | *R* = 0.66  *P* = 0.001 | *R* = 0.42  *P* = 0.051 | *R* = 0.56  *P* = 0.007 |
| **N of fanning events** | *R* = 0.82  *P* = 0.000 | *R* = -0.45  *P*= 0.0003 | | -- | *R* = 0.76  *P* = 0.000 | *R* = 0.69  *P* = 0.000 |
| **N of gluings** | *R* = 0.82  *P* = 0.000 | *R* = 0.76  *P* = 0.000 | | *R* = 0.764  *P* = 0.000 | -- | *R* = 0.58  *P* = 0.004 |
| **N of entrance showings** | *R* = 0.66  *P* = 0.001 | *R* = 0.76  *P* = 0.000 | | *R* = 0.69  *P* =0.000 | *R* = 0.58  *P* = 0.004 | -- |

**Table S2.** Full and final structural equation models with corrected Akaike Information Criteria (AIC). To evaluate model fit, we used AIC and the p-value for each model (*P* > 0.05 indicates adequate model fit). Tested paths are reaching-task score (Task), nest bulk area (Bulk), relative red area (Red), courtships (PC1 and PC2). Significant *P*-values are highlighted in bold.

|  |  | **Full model**  (AIC, 47.46; Fisher’s C, 15.46) | | **Final model**  (AIC, 13.95; Fisher’s C, 2.77) | |
| --- | --- | --- | --- | --- | --- |
| **Response variable** | **Paths tested** | **Estimate ± SE** | ***P*** | **Estimate ± SE** | ***P*** |
| Attractiveness | Task  Bulk  Red  PC1  PC2 | 0.467 ± 0.126  0.282 ± 0.128  0.021 ± 0.147  0.243 ± 0.094  0.078 ± 0.201 | 0.095  0.322  0.945  0.252  0.765 | 0.566±0.088 | **0.006** |
| Bulk  Red*  PC1  PC2 | Task  Task  Task  Task | 0.492 ± 0.201  -0.618^*^  -0.112 ± 0.230  0.009 ± 0.237 | **0.020**  **0.002**  0.620  0.968 | 0.492 ± 0.201  -0.618^*^ | **0.020**  **0.002** |
|  |  |  | 0.217 |  | 0.378 |

^*^Path was modelled as correlated errors, hence no standard errors for coefficients were estimated.
